# Broad-scale distribution of marine benthic litter in shallow waters along the Turkish Aegean coast: a SCUBA-based assessment

**DOI:** 10.1007/s11356-026-37630-1

**Published:** 2026-03-17

**Authors:** Yaşar Özvarol, Barış Akçalı, Erhan Mutlu

**Affiliations:** 1 Maritime Faculty, Akdeniz University, Campus, Antalya, Turkey; 2 Department of Marine Sciences, Institute of Marine Sciences and Technology, Dokuz Eylül University, Campus, İzmir, Turkey; 3 Fisheries Faculty, Akdeniz University, Campus, Antalya, Turkey

**Keywords:** Marine litter, Aegean Sea, SCUBA-assisted survey, Benthic macro debris, Mass–abundance metrics, Coastal management

## Abstract

**Purpose:**

Marine litter, particularly benthic macro debris, poses persistent ecological and socio-economic threats to Mediterranean seafloor ecosystems. This study addresses critical knowledge gaps in the spatial distribution, composition, and accumulation characteristics of benthic litter along the understudied Turkish Aegean coastline.

**Methods:**

We conducted the first large-scale, depth-stratified SCUBA-assisted assessment of benthic macro litter across 326 stations along approximately 1600 km of the Turkish Aegean coast. Surveys were performed at six discrete depth levels (5–30 m) following MSFD and UNEP/MAP protocols. Litter items were classified into nine standardized material categories, and both abundance (n km^−2^) and mass (kg km^−2^) metrics were quantified. Multivariate statistical analyses (PCA and PERMANOVA) were applied to examine spatial and depth-related patterns in litter distribution.

**Results:**

A total of 822 litter items (4147 kg) were recorded, corresponding to mean values of 77.4 n km^−2^ and 383 kg km^−2^. Plastics dominated numerical abundance but contributed relatively little to total mass, whereas metal and concrete debris accounted for a disproportionate share of mass. Shallow stations (5–10 m) were characterized by higher abundances of lightweight litter associated with nearshore activities, while deeper stations more frequently contained heavier debris linked to maritime and aquaculture-related operations. Spatial variability in litter accumulation was primarily associated with material composition and localized human activities rather than broad administrative divisions.

**Conclusion:**

This study provides the first harmonized and policy-relevant baseline for benthic litter along the Turkish Aegean coast. The findings highlight the importance of integrating mass-based indicators alongside abundance metrics to better assess ecological risk and inform management strategies. SCUBA-assisted surveys are shown to be a robust and complementary approach for monitoring benthic litter in shallow, heterogeneous coastal environments within MSFD/UNEP-aligned frameworks.

**Supplementary Information:**

The online version contains supplementary material available at 10.1007/s11356-026-37630-1.

## Introduction

Marine litter has become a major and multidimensional environmental challenge, with well-documented impacts on marine biodiversity, ecosystem functioning, and coastal economies worldwide (UNEP 2021). Among its various forms, benthic macro litter—defined as anthropogenic items larger than 2.5 cm deposited on the seafloor—represents a particularly persistent and less visible component of marine pollution. Its distribution is strongly influenced by bathymetry, seabed characteristics, and hydrodynamic energy gradients, which promote accumulation in depositional environments such as sheltered bays, port basins, seagrass meadows, and submarine canyons (Angiolillo and Fortibuoni 2020; Consoli et al. 2018; Cau et al. 2024).

The Mediterranean Sea is widely recognized as a global hotspot for marine litter accumulation due to its semi-enclosed configuration, dense coastal population, intense tourism, and high maritime traffic (Suaria and Aliani 2014; Boucher and Bilard 2020). Recent modelling and observational studies estimate that more than 229,000 tons of plastic enter the basin annually, with spatially heterogeneous retention and deposition patterns shaped by coastal morphology and circulation processes (Madricardo et al. 2020; Hernández et al. 2022; Le et al. 2024). High densities of benthic litter have been reported from several western and central Mediterranean regions, including the Ligurian Sea, the Balearic Islands, and the Sardinian Channel (Alomar et al. 2020; Angiolillo et al. 2021; Carreras-Colom et al. 2024). These studies consistently demonstrate that while plastics dominate item abundance, heavier materials such as metals, ceramics, and rubber contribute disproportionately to total mass and potential ecological impact (Olguner et al. 2018; Blanco et al. 2025).

In contrast, the eastern Mediterranean—and particularly the Turkish Aegean coastline—remains comparatively underrepresented in regional assessments of benthic litter, especially in very shallow coastal waters. Existing studies in Turkey have primarily focused on the Black Sea (Topçu et al. 2013; Eryaşar et al. 2014; Erüz et al. 2022; Özşeker et al. 2024; Terzi et al. 2025), the Sea of Marmara (Yenici and Türkoğlu 2022; Balcıoğlu İlhan 2025), and the eastern Mediterranean coast (Çevik et al. 2013; Güven et al. 2017; Gündoğdu 2017; Gündoğdu et al. 2020); Mutlu et al. 2020). Studies conducted in the Aegean Sea are largely limited to localized beach surveys or opportunistic trawl-based assessments, often lacking standardized depth stratification or mass-based metrics (Cerim et al. 2014; Öztekin and Bat 2017; Yılmaz et al. 2021).

As a result, many existing datasets remain methodologically inconsistent with monitoring protocols endorsed by the Marine Strategy Framework Directive (MSFD 2008 and the UNEP/MAP Regional Action Plan (UNEP/MAP 2015), limiting their suitability for integrated spatial analysis and policy evaluation (Galgani et al. 2013; Plastic Busters MPAs Project 2022). This inconsistency is particularly evident in relation to MSFD Descriptor 10 requirements. Several datasets fail to apply standardized depth stratification (UNEP/MAP 2015), omit mass-based metrics (MSFD 2008; Galgani et al. 2013), or adopt classification schemes that diverge from UNEP/MAP recommendations (UNEP/MAP 2015; Plastic Busters MPAs Project 2022). For instance, some studies report only item abundance without weight-based measures, while others group materials inconsistently (e.g., plastics, metals, ceramics, and rubber under varying categories). Such methodological divergence reduces comparability, complicates regional synthesis, and constrains the policy relevance of existing data.

Methodological heterogeneity therefore represents a major obstacle to the synthesis and comparison of benthic litter data across the Mediterranean. Differences in survey platforms, spatial resolution, habitat coverage, and litter classification schemes directly influence detectability, reporting, and cross-regional comparability under MSFD Descriptor 10 (Galgani et al. 2013). While the MEDIST framework provides standardized guidance for trawl-based surveys and has been widely applied in several Mediterranean studies (Olguner et al. 2018; Mutlu et al. 2020; Yenici and Türkoğlu 2022; Şirin et al. 2022), its applicability is limited in shallow and structurally complex coastal environments.

Much of the Aegean coastline is characterized by rocky or mixed substrates, irregular bathymetry, and nearshore habitats where trawling is either impractical or ecologically undesirable. Under these conditions, SCUBA-assisted surveys represent a particularly effective and innovative approach to benthic litter monitoring, enabling direct observation and collection in environments that are otherwise poorly accessible to conventional sampling platforms. By integrating standardized depth stratification (5–30 m across six intervals) and harmonized classification schemes aligned with MSFD and UNEP/MAP protocols, this approach allows high-resolution, item-level assessments of seabed litter in complex coastal habitats while remaining compatible with existing regional monitoring frameworks.

To address these knowledge and methodological gaps, the present study delivers the first depth-stratified, SCUBA-assisted, large-scale assessment of benthic macro litter along the entire Turkish Aegean coastline, extending from the Saros Gulf in the north to the Muğla–Mediterranean boundary in the south. Visual surveys were conducted at 326 sampling stations across six depth intervals (5–30 m), integrating both abundance (n km⁻^2^) and mass (kg km⁻^2^) metrics in line with MSFD Descriptor 10 recommendations (MSFD 2008; UNEP/MAP 2015). By combining extensive spatial coverage with a harmonized methodological framework, this study provides a policy-relevant baseline for evaluating the distribution, composition, and potential drivers of benthic litter in one of the Mediterranean’s most socio-ecologically diverse coastal regions.

## Materials and methods

### Study area and sampling framework

The study was conducted along the Turkish Aegean coastline, extending from the Saros Gulf in the north to the Muğla–Mediterranean boundary in the south. Sampling stations were selected using a stratified design that integrated coastal geomorphology (rocky versus sandy substrates, sheltered versus exposed coasts), proximity to major anthropogenic pressure sources (urban centers, aquaculture facilities, industrial harbors, and tourism hotspots), and operational feasibility for SCUBA diving.

**Fig. 1:**
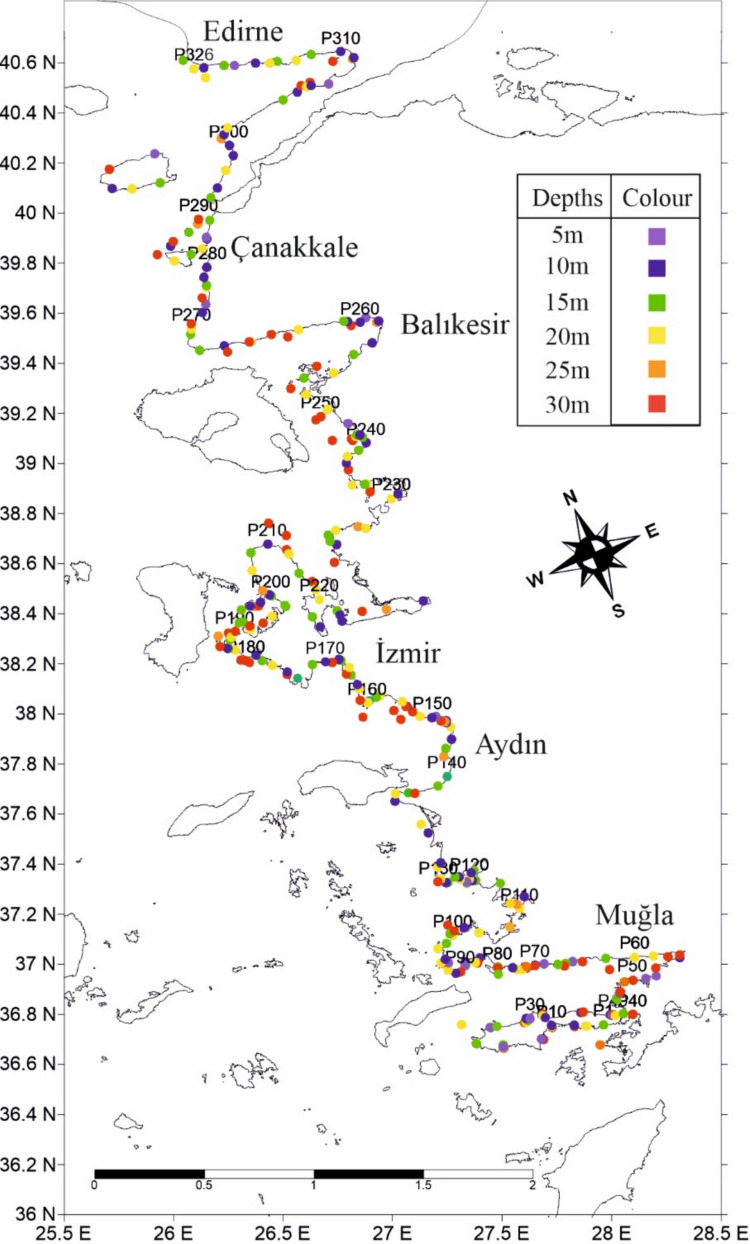
Map of sampling stations surveyed by diving in the study. The depth of each station is indicated with a different color

A total of 326 stations (Fig. 1) were surveyed across six coastal provinces, proportionally distributed according to shoreline length, depth availability, and logistical accessibility: Muğla (*n* = 134), İzmir (*n* = 99), Çanakkale (*n* = 36), Balıkesir (*n* = 30), Aydın (*n* = 22), and Edirne (*n* = 5). Surveys were conducted at six predefined depth intervals (5, 10, 15, 20, 25, and 30 m), selected to represent near-coastal depositional environments most affected by human activities while remaining within safe SCUBA operational limits (Galgani et al. 2013; Plastic Busters MPAs Project 2022).

This depth range encompasses the zone where litter inputs from tourism, small-scale fisheries, anchoring, aquaculture operations, and coastal infrastructure are most likely to accumulate before being redistributed to deeper environments.

### SCUBA-assisted field surveys

Fieldwork was conducted during summer 2024 (June–August) aboard the research vessel *R/V Akdeniz Su*. Survey depths were established prior to each dive based on vessel-mounted echosounder measurements and verified using analog pressure gauges. Each sampling station was therefore defined by a single target bottom depth (5, 10, 15, 20, 25, or 30 m). At the selected depth, a 10 × 10 m (100 m^2^) sampling area was delineated on the seabed, within which all macrobenthic litter was recorded and collected by two experienced scientific divers. Sampling was conducted at discrete stations, with each station representing an independent depth-specific observation. This procedure was applied consistently across all six depth levels throughout the study area.

Underwater visibility conditions were suitable for visual surveys across the study area. To characterize light availability at depth, photosynthetically active radiation (PAR) reaching the seabed was measured at each station using a LI-COR LI-192 underwater quantum sensor connected to a multirecorder (Fig. S1). Bottom-level irradiance values ranged from 3.243 to 6.261 µmol photons m⁻^2^ s⁻^1^, indicating relatively stable optical conditions within the surveyed depth range.

The sampling area was delineated underwater using interconnected 10-m measuring rods arranged to form a modular square, allowing consistent area definition across heterogeneous seabed morphologies. This approach ensured methodological standardization while maintaining flexibility in rocky and irregular habitats.

SCUBA-based surveys were particularly suited to the Turkish Aegean coast, where steep slopes, rocky substrates, and abrupt depth gradients limit the applicability of trawl-based methods and complicate ROV operations. Direct diver observations enabled close inspection of irregular surfaces, crevices, and partially buried features, facilitating the detection of small plastic items, entangled fishing gear, aquaculture-related debris, and other litter types that may be overlooked by remote techniques.

All movable litter within each sampling unit was collected, classified, and weighed. Items of cultural or archeological relevance were recorded but left in situ in accordance with national heritage regulations. For large or immobile objects (e.g., tires, anchors, concrete blocks), dimensions were measured underwater and mass was estimated using reference density tables and established visual estimation procedures (Cau et al. 2024; Yılmaz et al. 2021), ensuring that non-retrievable items were consistently incorporated into abundance and mass estimates.

### Litter classification and metric standardization

All collected litter items were classified into nine material categories following UNEP/MAP, MSFD, and GESAMP typologies to ensure cross-regional comparability (MSFD 2008; GESAMP 2019; UNEP 2021) (Appendix 1, Table S1). Composite objects were assigned to a single category based on the dominant material, determined by relative volume and weight.

Item counts recorded within each 100 m^2^ sampling unit were standardized to number per square kilometer (n km⁻^2^) by multiplying by 10,000, in accordance with MSFD Descriptor 10 reporting standards (MSFD Technical Subgroup on Marine Litter 2013).

Mass was measured as weight in grams, converted to kilograms, and similarly scaled to kg km⁻^2^ using the following relationship:

$$\mathrm{Mass}\;(\text{kg km}^{-2}) = (\text{g per 100 m}^2) \times 10$$

Mass was recorded as dry weight to reduce variability associated with moisture content and to ensure consistency with MSFD and UNEP/MAP protocols as well as comparability with regional Mediterranean datasets. For this purpose, all collected litter items were air-dried at room temperature prior to weighing, and measurements were taken only after a constant weight was achieved through successive weighings. Drying duration was extended for water-absorbent materials such as textiles and sponges to ensure the complete removal of free water. This approach facilitates reliable mass standardization and integration with basin-scale assessments across Mediterranean sub-basins (Olguner et al. 2018; Carreras-Colom et al. 2024; Dağtekin et al. 2025).

### Statistical analyses

Multivariate analyses were applied to examine patterns in benthic litter distribution across depth and space using both abundance (n km⁻^2^) and mass (kg km⁻^2^) data. Mean abundance (n km⁻^2^) and mass (kg km⁻^2^) values were calculated using the complete dataset, including both litter-positive stations and stations where no benthic litter was detected (zero values). This approach was applied consistently in depth-stratified and province-based summaries.

Principal component analysis (PCA) was used to explore major gradients in litter composition and to visualize relationships among sampling stations based on material categories and depth. Separate PCAs were conducted for abundance and mass to account for differences between item counts and material weight.

Differences in litter composition were further tested using a two-way permutational multivariate analysis of variance (PERMANOVA), with depth and province included as fixed factors. Analyses were based on Euclidean distance matrices and 999 permutations, with Monte Carlo *p*-values applied where appropriate.

All statistical analyses were performed using PRIMER v6 with the PERMANOVA + add-on (Clarke and Gorley 2015).

## Results

### General overview of benthic litter distribution

**Fig. 2:**
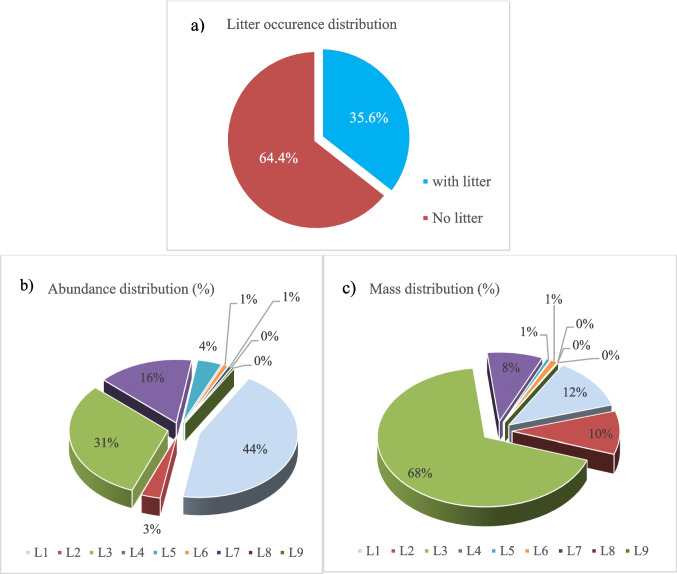
**a** Number of stations with and without benthic macro litter. **b** Percentage composition by abundance (n km^−2^). **c** Percentage composition by mass (kg km^−2^). Note: L1, plastic; L2, rubber; L3, metal; L4, glass/ceramic/concrete; L5, textiles/natural fibers; L6, processed wood; L7, paper/cardboard; L8, other; L9, unspecified. Categories apply to all figures unless otherwise stated

Across the 326 SCUBA-surveyed stations along the Turkish Aegean coastline, a total of 822 benthic litter items were documented, with a combined mass of 4147 kg. This corresponds to a mean abundance of 77.4 ± 4.6 n km⁻^2^ and a mean mass of 383 ± 43 kg km⁻^2^. Benthic litter was recorded at 116 stations, accounting for 35.6% of the total surveyed sites (Fig. 2a).

### Material composition patterns

Marine litter composition differed between numerical abundance and mass contribution. Plastics (L1) were the most frequently recorded material group, comprising 44.4% of the total number of items, while contributing 11.9% of the total mass (493.6 kg). Metals (L3) accounted for 31.0% of recorded items and represented 67.6% of the total mass (2802.8 kg), including items such as anchors, chains, and other metal debris.

Other material groups, including glass/ceramic/concrete (L4), rubber (L2), and textiles/natural fibers (L5), contributed to both abundance and mass in varying proportions (Fig. 2b, c). Concrete ballast classified under subcategory L4d contributed 212.0 kg to the total mass, corresponding to 19.9% of the overall weight.

### Depth-related stratification

**Fig. 3:**
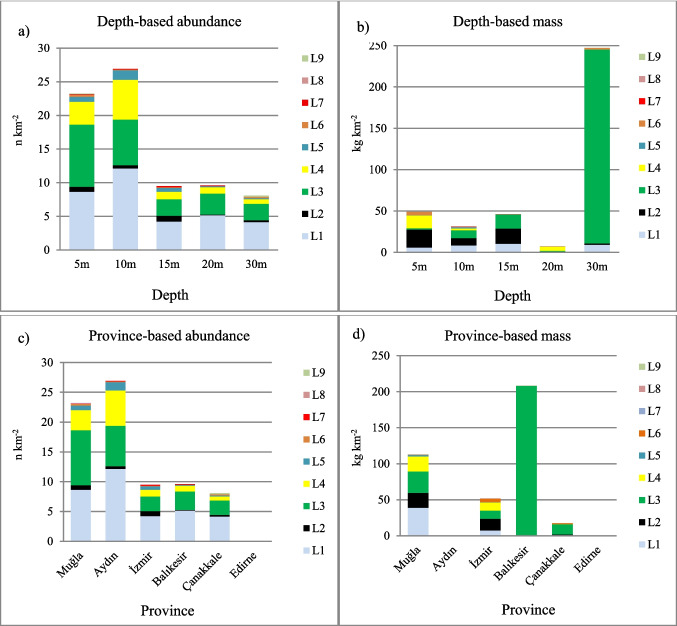
**a** Abundance of benthic litter by depth (n km^−2^). **b** Mass of benthic litter by depth (kg km^−2^). **c** Abundance of benthic litter by province (n km^−2^). **d** Mass of benthic litter by province (kg km^−2^). Litter categories (L1–L9) are defined according to the UNEP/MAP–MSFD classification scheme and are described in detail in Fig. 2

Depth-stratified analyses showed different distributions of numerical abundance and mass across sampled depth intervals. The highest numerical abundance values were recorded at shallow depths, with 48.8 n km⁻^2^ at 5 m and 57.2 n km⁻^2^ at 10 m. In contrast, mass values increased with depth, reaching 523.1 kg km⁻^2^ at 30 m, compared to 71.3 kg km⁻^2^ at 5 m (Fig. 3a, b).

### Spatial distribution and composition of benthic litter

Benthic litter abundance and mass were quantified across coastal sectors of the Turkish Aegean Sea. When depth strata were evaluated together with provincial boundaries, mass values differed among provinces and depth intervals. In Muğla, litter mass was recorded at all sampled depths, with values of 10.6 kg km⁻^2^ at 5 m and 35.0 kg km⁻^2^ at 30 m. In İzmir, mass values reached 23.0 kg km⁻^2^ at 5 m. In Balıkesir, a mass value of 208.4 kg km⁻^2^ was recorded at 30-m depth. In Aydın and Çanakkale, mass values remained below 15 kg km⁻^2^ across all sampled depths. No benthic litter was recorded at stations surveyed in Edirne.

**Fig. 4:**
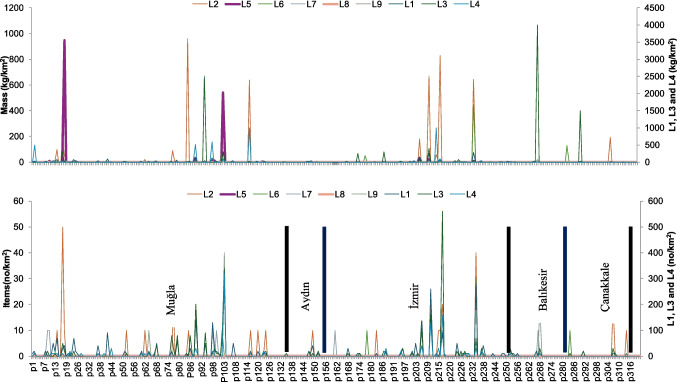
Spatial variation in benthic litter abundance (n km⁻^2^) and mass (kg km⁻^2^) across coastal sectors of the Turkish Aegean Sea. Bars represent mean abundance values, while symbols indicate corresponding mean mass values for each coastal sector. Litter categories (L1–L9) follow the UNEP/MAP–MSFD classification scheme and are described in detail in Fig. 2

Mean numerical abundance and mass values were calculated by including all surveyed stations within each coastal sector, including stations where no benthic litter was detected (zero values). Of the 134 sampling stations, benthic litter was recorded at 49 stations (36.6%) in Muğla and 31 stations (31.3%) in İzmir. Detection frequencies were 27.3% in Aydın, 23.3% in Balıkesir, and 13.9% in Çanakkale. Mean numerical abundance values were 90.4 n km⁻^2^ in Muğla, 63.2 n km⁻^2^ in İzmir, and 3.4 n km⁻^2^ in Aydın. Mean mass values were 443.0 kg km⁻^2^ in Balıkesir, 239.6 kg km⁻^2^ in Muğla, and 109.6 kg km⁻^2^ in İzmir. Mean mass values in Aydın and Çanakkale were below 15 kg km⁻^2^ (Fig. 3c, d; Fig. 4).

Numerical abundance and mass-based metrics differed among coastal sectors (Fig. 3c, d; Fig. 4). Higher numerical abundance values were recorded at stations in Muğla and İzmir, whereas stations in Balıkesir exhibited lower abundance values and higher mass values.

Recorded benthic litter items varied among coastal sectors. At stations in Muğla, items included plastic bottles, diving equipment, beach-related objects, tires, and lead batteries. At stations in İzmir, recorded items included metal fragments, buoys, and concrete blocks. Nets and monofilament lines were recorded at stations in Balıkesir and Aydın. At stations in Çanakkale, recorded items included amphorae, anchors, chain links, and concrete ballast blocks. Concrete ballast blocks, with a mean item weight of 42.4 kg, were recorded at 12 stations.

### Multivariate patterns in litter composition

Principal component analysis (PCA) was applied to standardized station-level abundance and mass data to examine multivariate patterns in benthic litter composition. PCA ordination plots for abundance and mass data are shown in Fig. A1 and Fig. A2, respectively.

The PCA ordinations did not show distinct clustering of sampling stations according to provincial groupings or bottom depth categories in either the abundance-based or mass-based analyses (Fig. A1a–b; Fig. A2a–b).

Results of the two-way PERMANOVA analysis showed no significant main effect of city on litter abundance (*p* = 0.327; p(MC) = 0.75) or mass (*p* = 0.389; p(MC) = 0.79) (Table A3). Bottom depth also showed no significant main effect on abundance or mass (Table A3). The interaction between city and depth was not significant for abundance, while a marginally significant interaction effect was detected for mass based on Monte Carlo permutation results (p(MC) = 0.038) (Table A3).

## Discussion

This study presents the first harmonized, SCUBA-assisted large-scale assessment of benthic macro litter along the Turkish Aegean coast, integrating both abundance and mass metrics across 326 stations. The detection rate of 35.6% falls within the range reported for semi-enclosed Mediterranean basins (20–50%), where litter occurrence is modulated by hydrodynamic conditions and the spatial concentration of coastal activities (Galgani et al. 2000; Suaria and Aliani, 2014; Carreras-Colom et al. 2024). Comparable accumulation patterns have been documented in Haifa Bay and the Balearic Islands, where elevated litter levels are linked not solely to item counts but to the combined effect of material composition, mass, and persistent anthropogenic pressures such as tourism, aquaculture, and port operations (Alomar et al. 2020; Le et al. 2024).

In the present study, stations exposed to industrial, aquaculture, and recreational activities consistently exhibited higher litter abundance accompanied by disproportionately elevated mass. This pattern mirrors observations from the Saronikos Gulf and the Tunisian coastline, reinforcing the notion that accumulation hotspots are therefore better characterized by qualitative attributes—particularly mass and material composition—rather than by numerical density alone. (Kouvara et al. 2024; Cau et al. 2024).

Beyond detection frequency, the magnitude of benthic litter accumulation along the Turkish Aegean coast was notable. Mean mass (383 kg km^−2^) and abundance (77.4 n km^−2^) exceeded values reported from earlier SCUBA-assisted surveys along the Muğla–Antalya coastline (Olguner et al. 2018) and were higher than those derived from trawl-based studies in the Sea of Marmara and Iskenderun Bay (Gündoğdu et al. 2020; Şirin et al. 2022). Comparable densities have been reported in Mediterranean regions characterized by intensive coastal use, including the Gulf of Sant Jordi, the Ligurian Sea, and the Sardinian Channel (Consoli et al. 2018; Angiolillo et al. 2021; Carreras-Colom et al. 2024). These similarities underscore the central role of human-dominated coastal sectors—marinas, aquaculture facilities, shipyards, and tourism corridors—in shaping seabed litter accumulation.

Material-specific analyses revealed a clear divergence between numerical abundance and mass contribution. Plastics dominated in terms of item counts but accounted for a relatively small proportion of total mass, whereas metals and concrete elements contributed disproportionately to overall mass despite lower abundance. Similar imbalances have been reported in deeper and industrialized Mediterranean settings, where heavy debris strongly influences mass-based accumulation metrics (Angiolillo et al. 2021; Carreras-Colom et al. 2024).

Multivariate analyses did not reveal distinct clustering of sampling stations according to provincial boundaries or depth categories, as indicated by both PCA ordinations and PERMANOVA results. The lack of significant main effects for city and depth suggests that benthic litter composition is not structured by these factors alone but rather reflects heterogeneity at finer spatial scales. The marginal interaction between province and depth observed for mass-based data suggests that local depth-related processes may influence mass accumulation differently across regions, although this pattern was not evident for numerical abundance.

Mass-based analysis further revealed that aquaculture-derived debris—particularly concrete ballast materials and metal components—constituted a substantial fraction of total benthic mass in several high-impact zones. Similar patterns, in which heavy aquaculture-related materials disproportionately influence mass-based litter metrics, have been reported from other Mediterranean regions characterized by offshore production activities (Angiolillo et al. 2021; Carreras-Colom et al. 2024), confirming the regional significance of this issue. These materials pose dual ecological risks by contributing to physical abrasion of the seabed and by introducing long-lasting, chemically reactive substrates into benthic environments. Given their persistence and disproportionate contribution to mass accumulation, the results support the need for regulatory measures requiring the recovery or replacement of ballast materials during aquaculture facility decommissioning, in order to reduce long-term benthic disturbance.

Depth-related patterns provided additional context for these trends. Shallow waters (5–15 m) exhibited higher densities of lightweight debris, particularly plastics and fishing-related items such as nets and monofilament lines. Comparable depth-associated dominance of plastics has been reported in SCUBA-based surveys from Antalya and the Sea of Marmara (Olguner et al. 2018; Şirin et al. 2022). In contrast, deeper stations (> 15 m) more frequently contained heavier items, including metal objects, anchors, aquaculture buoys, and concrete ballast materials, as also observed in other Mediterranean studies (Madricardo et al. 2020; Cau et al. 2024).

These depth-related patterns are not readily attributable to density-driven passive deposition alone. Rather, they appear consistent with the spatial distribution of human activities across the coastal zone, where depth may act as a proxy for differing use regimes. The occurrence of substantial litter mass at shallow depths in certain locations further suggests that localized practices, such as anchoring or recreational boating, can influence seabed litter accumulation independently of simple hydrodynamic sorting processes (Alomar et al. 2020; Vlachogianni et al. 2019).

The pronounced spatial variability in benthic litter distribution observed in this study appears to be influenced by both the intensity of anthropogenic activities and local hydrodynamic conditions. Areas exhibiting higher litter abundance and mass frequently coincided with coastal sectors characterized by concentrated maritime uses, including marina infrastructure, aquaculture installations, and other coastal activities. Comparable associations between activity-related inputs and seabed litter accumulation have been reported for industrialized and aquaculture-influenced sectors of the eastern Aegean and adjacent Mediterranean regions (Cerim et al. 2014; Öztekin and Bat 2017; Gönülal et al. 2024; Vlachogianni et al. 2018).

Conversely, sites with low or absent litter occurrence tended to correspond to areas subject to stronger hydrodynamic exposure and comparatively lower levels of coastal use. Similar observations have been documented in other Mediterranean settings, where energetic circulation regimes are thought to limit local seabed accumulation by redistributing debris toward lower-energy depositional environments (Lebreton et al. 2012; Kaladharan et al. 2020; Madricardo et al. 2020).

From a methodological perspective, the results indicate the suitability of SCUBA-assisted surveys for assessing benthic litter in shallow, heterogeneous coastal environments. Unlike bottom trawling or ROV-based approaches, which are constrained by seabed morphology and often underestimate lighter or partially buried items, diver-based surveys allow direct visual confirmation, precise in situ measurements, and reliable estimation of both abundance and mass. This capability is particularly important in rocky, sloping, or structurally complex habitats where trawling is impractical or ecologically damaging (Galgani et al. 2013; Pham et al. 2014; Ioakeimidis et al. 2015). Comparable SCUBA-assisted applications in Greek waters further support the value of this approach for high-resolution coastal assessments (Kouvara et al. 2024).

The divergence between abundance and mass-based indicators observed in this study aligns with recent recommendations to include weight-sensitive metrics in marine litter monitoring. Mass-based analyses provided complementary information to numerical abundance, particularly by highlighting the contribution of heavier materials to localized accumulation patterns. Similar observations from other Mediterranean studies emphasize the relevance of mass-based metrics for characterizing benthic litter distributions and informing assessments of physical seabed impacts (Angiolillo and Fortibuoni 2020; Carreras-Colom et al. 2024).

The absence of detected benthic litter at stations surveyed in Edirne may be related to the comparatively limited intensity of coastal and maritime activities in the region, including the lack of large-scale tourism and yachting infrastructure, minimal aquaculture operations, limited fishing pressure, and the absence of major nearshore industrial facilities and maritime transport routes.

Finally, the detection of amphora fragments and other heritage-related materials underscores the need for heritage-sensitive management strategies in litter surveys. In archeologically rich areas such as the northern Aegean, environmental remediation efforts must be carefully balanced with cultural preservation, requiring interdisciplinary collaboration among marine ecologists, archeologists, and policymakers (Edvardsson et al. 2025).

## Conclusion

This study presents the first depth-stratified, SCUBA-assisted assessment of benthic macro-litter along the Turkish Aegean coastline, employing a dual-indicator framework that integrates item abundance and material mass across a broad spatial scale. By combining numerical and weight-based metrics, the analysis provides a more comprehensive characterization of benthic litter distribution, composition, and accumulation patterns than abundance-only approaches, establishing a robust and policy-relevant baseline for the eastern Mediterranean.

The pronounced mismatch between abundance and mass-based indicators demonstrates that the numerical dominance of plastics does not necessarily reflect benthic pressure, emphasizing the critical role of weight-sensitive metrics in marine litter assessments. While plastics were the most frequently encountered material across all surveyed sectors, heavier materials—particularly metals, rubber, and concrete elements—accounted for a substantial proportion of total mass, underscoring their disproportionate contribution to long-term seabed impact and physical disturbance.

Marked spatial and bathymetric heterogeneity was observed throughout the study area. Coastal sectors influenced by intensive tourism, aquaculture operations, fishing activities, and maritime traffic consistently emerged as accumulation hotspots. Shallow waters were primarily characterized by lightweight and buoyant debris associated with nearshore human activities, whereas deeper strata exhibited increased accumulation of dense materials such as anchors, concrete ballasts, and industrial remnants. These patterns indicate that benthic litter distribution is closely linked to the spatial footprint of human activities, with depth acting as a contextual modifier rather than an independent driver of large-scale spatial structuring.

The findings support the integration of mass-based indicators into existing monitoring frameworks, including the Marine Strategy Framework Directive (MSFD), UNEP/MAP Regional Action Plan, and GESAMP guidelines, to enhance the assessment of ecological risk and management effectiveness. In particular, aquaculture-related debris warrants targeted attention, as the widespread use of concrete ballasts contributes substantially to benthic mass accumulation and may induce persistent physical impacts on seabed habitats. Transitioning toward retrievable or alternative anchoring systems represents a practical pathway to mitigate long-term benthic disturbance.

Overall, this study delivers a scientifically robust and harmonized baseline for benthic litter monitoring in shallow, heterogeneous coastal environments and demonstrates the value of SCUBA-assisted surveys as a complementary tool to conventional sampling approaches. The results provide critical evidence to support spatially targeted, material-specific management strategies aligned with regional marine environmental policies and the objectives of United Nations Sustainable Development Goal 14.

## Supplementary Information

Below is the link to the electronic supplementary material.

ESM 1 (DOCX 448 KB)

## Data Availability

The datasets generated and/or analyzed during the current study are not publicly available but will be made available by the corresponding author upon reasonable request.
