# Correction to: Broad-scale distribution of marine benthic litter in shallow waters along the Turkish Aegean coast: a SCUBA-based assessment

**DOI:** 10.1007/s11356-026-37776-y

**Published:** 2026-05-01

**Authors:** Yaşar Özvarol, Barış Akçalı, Erhan Mutlu

**Affiliations:** 1 Maritime Faculty, Akdeniz University, Campus, Antalya, Turkey; 2 Department of Marine Sciences, Institute of Marine Sciences and Technology, Dokuz Eylül University, Campus, İzmir, Turkey; 3 Fisheries Faculty, Akdeniz University, Campus, Antalya, Turkey

**Correction to: Environmental Science and Pollution Research (2026) 33:5121-5132**

10.1007/s11356-026-37630-1

In the Results section under General overview of benthic litter distribution, the caption of Figure 2 is repeated and should be removed.

The Original article has been corrected.

