# Supplementary material for: Broad-scale distribution of marine benthic litter in shallow waters along the Turkish Aegean coast: a SCUBA-based assessment
Source: Environ Sci Pollut Res Int. 2026 Mar 17;33(11):5121–32. doi: 10.1007/s11356-026-37630-1 (PMC13056772; doi:10.1007/s11356-026-37630-1)
Supplement: Supplementary file 1 — (DOCX 448 KB) [file 11356_2026_37630_MOESM1_ESM.docx]

**
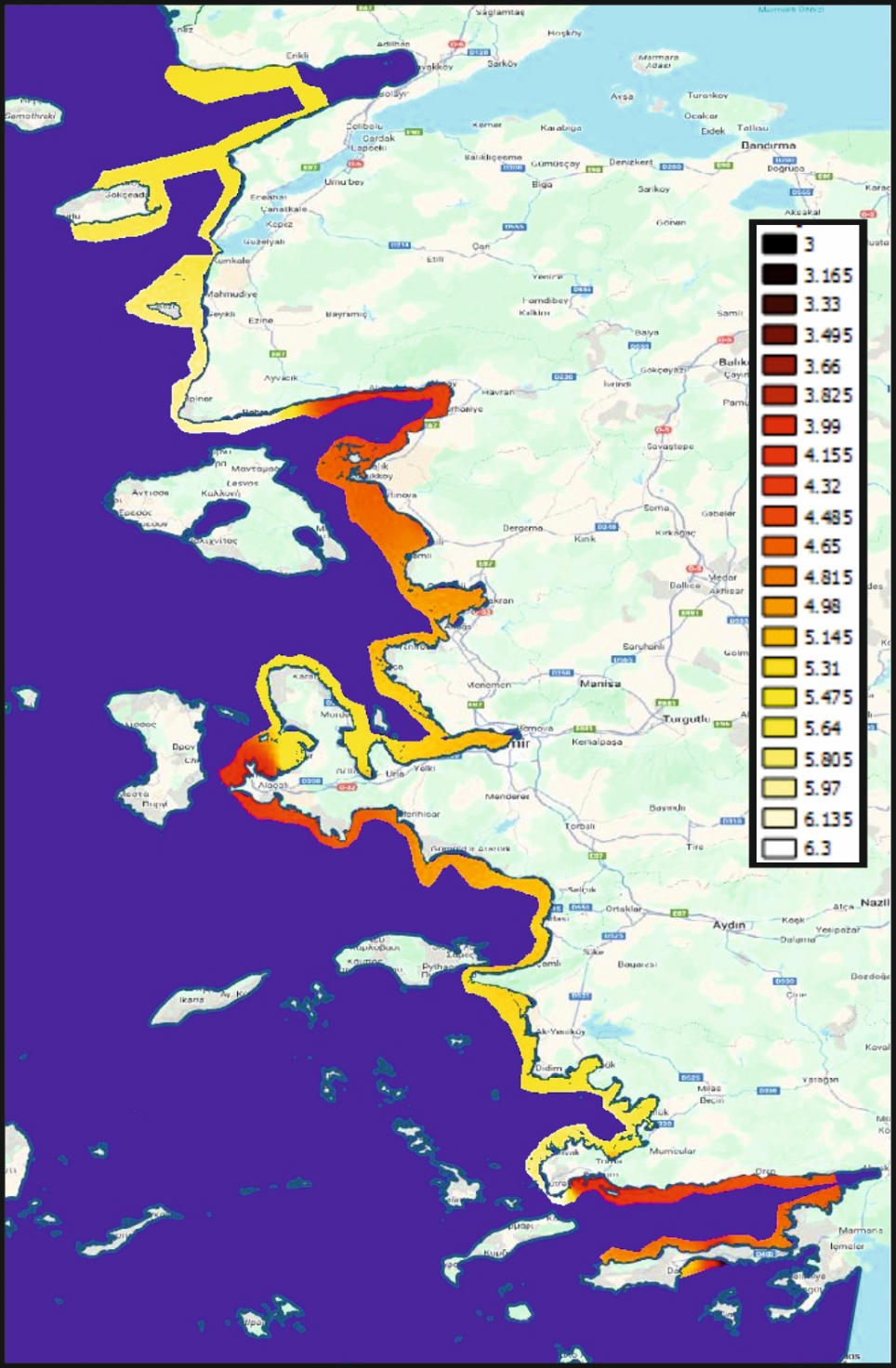
**

**Fig S1**. Intensity of photosynthetically active radiation (PAR) reaching the seabed at survey depths (5–30 m) along the Turkish Aegean coast. Measurements were obtained with a LI-COR LI-192 underwater irradiance meter and indicate sufficient water transparency for visual detection of benthic macro-litter at all stations. Red dashed area shows marine protected area (MPA) (from Mutlu 2025)

**Appendix**

**Table A1**. Total mass (TW; kg), mean (MW; kg km^-2^) number (TN; n) and mean (MN; n km^-2^) of individual and of the litter categorized (some added to L9 Unspecified or uncategorized) according to typology by MEDIST (Mediterranean Depth-Integrated Survey Technique) in coastal waters in the study area.

| **Typology** | **TW** | **MW** | **TN** | **MN** |
| --- | --- | --- | --- | --- |
| **L1 Plastic (including PVC, polypropylene, polyethylene)** | 493.6 | 46.3 | 366 | 34.4 |
| L1a. Bags | 0.7 | 0.1 | 122 | 11.5 |
| L1b. Bottles | 7.4 | 0.6 | 116 | 10.9 |
| L1c. Food wrappers | 0 | 0 | 6 | 0.6 |
| L1d. Sheets (table-cover, etc.) | 1.9 | 0.2 | 12 | 1.1 |
| L1e. Hard plastic objects (crates, containers, tubes, ash-trays, lids, etc.) | 158.1 | 14.9 | 68 | 6.4 |
| L1f. Fishing nets | 14.9 | 1.4 | 3 | 0.3 |
| L1g. Fishing lines | 0.2 | 0 | 5 | 0.5 |
| L1h. Other fishing related (pots, floats, etc.) | 302.4 | 28.4 | 20 | 1.9 |
| L1i. Synthetic ropes/strapping bands | 3.5 | 0.3 | 11 | 1 |
| L1j. others (plastic cup) | 4.5 | 0.4 | 3 | 0.3 |
| **L2 Rubber** | 412.9 | 38.8 | 24 | 2.3 |
| L2a. Tyres | 410.6 | 38.6 | 18 | 1.7 |
| L2b. Other (gloves, floats, boots/shoes, oilskins, sanitaries) | 2.3 | 0.2 | 6 | 0.6 |
| **L3 Metal** | 2802.8 | 263.7 | 260 | 24.5 |
| L3a. Beverage cans | 8.5 | 0.8 | 218 | 20.5 |
| L3b. Other food cans/wrappers | 0 | 0 | 3 | 0.3 |
| L3c. Middle size containers (of paint, oil, chemicals) | 4.9 | 0.5 | 12 | 1.1 |
| L3d. Large metalic objects (barrels, pieces of machinery, electric appliances) | 2535.9 | 238.6 | 10 | 0.9 |
| L3e. Cables | 0 | 0 | 1 | 0.1 |
| L3f. Fishing related (hooks, spears, etc. Includes aquaculture gear) | 253.5 | 23.9 | 16 | 1.5 |
| L3g. remnant from the war | 0 | 0 | 0 | 0 |
| **L4 Glass / Ceramic/Concrete** | 343.5 | 32.3 | 123 | 11.6 |
| L4a. Bottles | 34 | 3.2 | 106 | 10 |
| L4b. Pieces of glass | 0.9 | 0.1 | 8 | 0.8 |
| L4c. Ceramic jars, plates and cups | 96.5 | 9.1 | 4 | 0.4 |
| L4d. Large objects (ceramic basins, etc (include Aquaculture ballasts)) | 212 | 19.9 | 5 | 0.5 |
| **L5 Cloth (textile) / Natural fibers** | 26.2 | 2.5 | 35 | 3.3 |
| L5a. Clothing (clothes, shoes, etc.) | 14.2 | 1.3 | 24 | 2.3 |
| L5b. Large pieces (carpets, mattresses, etc.) | 11.6 | 1.1 | 11 | 1 |
| L5c. Natural ropes | 0.4 | 0 | 0 | 0 |
| L5d. Sanitaries (diapers, cotton buds, etc.) | 0 | 0 | 0 | 0 |
| **L6 Wood processed (palettes, crates, etc.)** | 67.6 | 6.4 | 6 | 0.6 |
| **L7 Paper and cardboard** | 0.3 | 0 | 5 | 0.5 |
| **L8 Other** | 0 | 0 | 0 | 0 |
| **L9 Unspecified (uncategorized)** | 2.2 | 0.2 | 3 | 0.3 |
| Ancient litter | 2.22 | 0.02 | 3 | 0.03 |

**Table A2.** Total mass (TW; kg) and number (TN; n) of individual and of the litter categorized according to Marine Strategy Framework Directive (MSFD, 2008) in coastal waters in the study area

| **J-Code** | **SUP/FG** | **Name** | **Count** | **Weight (Kg)** |
| --- | --- | --- | --- | --- |
|  |  | **Artificial Polymer Materials** |  |  |
| J220 |  | Plastic sheeting from greenhouses | 0 | 0.0 |
| J221 |  | Plastic irrigation pipes | 7 | 28.9 |
| J222 |  | Other plastic items from agriculture | 0 | 0.0 |
| J90 |  | Plastic flower pots | 0 | 0.0 |
| J223 |  | Trays for seedlings of foamed plastic | 0 | 0.0 |
| J46 | FG | Plastic oyster trays | 0 | 0.0 |
| J45 | FG | Plastic mussels/oyster mesh bags, net sack, socks | 0 | 0.0 |
| J47 | FG | Plastic sheeting from mussel culture (Tahitians) | 0 | 0.0 |
| J102 |  | Plastic flip-flops | 0 | 0.0 |
| J136 |  | Footwear made of plastic not flip flops | 0 | 0.0 |
| J40 |  | Plastic gloves (household/dishwashing, gardening) | 0 | 0.0 |
| J41 |  | Plastic gloves (industrial/professional applications) | 0 | 0.0 |
| J252 |  | Single-use plastic gloves | 0 | 0.0 |
| J69 |  | Plastic hard hats/helmets | 0 | 0.0 |
| J256 |  | Foamed plastic insulation including spray foam | 0 | 0.0 |
| J89 |  | Plastic construction waste (not foamed insulation) | 0 | 0.0 |
| J8 | SUP | Plastic drink bottles >0.5 l | 110 | 6.0 |
| J7 | SUP | Plastic drink bottles <0.5 l | 6 | 0.4 |
| J224 | SUP | Plastic food containers made of foamed polystyrene | 0 | 0.0 |
| J21 * | SUP | Plastic caps/lids drinks | 0 | 0.0 |
| J225 | SUP | Plastic food containers made of hard non-foamed plastic | 0 | 0.0 |
| J1 | SUP | Plastic 4/6-pack yokes & six-pack rings | 0 | 0.0 |
| J226 | SUP | Cups and cup lids of foamed polystyrene | 0 | 0.0 |
| J227 | SUP | Cups and lids of hard plastic | 0 | 0.0 |
| J228 | SUP | Plastic cutlery | 0 | 0.0 |
| J229 | SUP | Plastic plates and trays | 3 | 1.2 |
| J230 | SUP | Plastic stirrers | 0 | 0.0 |
| J231 | SUP | Plastic straws | 0 | 0.0 |
| J30 | SUP | Plastic crisps packets/sweets wrappers | 6 | 0.0 |
| J31 | SUP | Plastic lolly & ice-cream sticks | 0 | 0.0 |
| J85 | FG | Plastic commercial salt packaging | 0 | 0.0 |
| J58 | FG | Fish boxes foamed polystyrene | 0 | 0.0 |
| J57 | FG | Fish boxes hard plastic | 0 | 0.0 |
| J92 | FG | Plastic bait containers/packaging | 0 | 0.0 |
| J60 * | FG | Plastic fishing light sticks/fishing glow sticks incl. Packaging | 0 | 0.0 |
| J62 | FG | Plastic floats for fishing nets | 0 | 0.0 |
| J59 | FG | Plastic fishing line | 2 | 0.1 |
| J54 | FG | Plastic nets and pieces of net >50cm | 3 | 14.9 |
| J53 | FG | Plastic nets and pieces of net <50 | 0 | 0.0 |
| J232 | FG | Plastic string and filaments exclusively from dolly ropes | 0 | 0.0 |
| J233 | FG | Other plastic string and filaments exclusively from fishery | 0 | 0.0 |
| J234 | FG | Plastic tangled nets and rope without dolly rope or mixed with dolly rope | 0 | 0.0 |
| J235 | FG | Plastic tangled dolly rope | 0 | 0.0 |
| J61 | FG | Other plastic fisheries related items not covered by other categories | 17 | 298.6 |
| J42 | FG | Plastic crab/lobster traps (pots) and tops | 0 | 0.0 |
| J44 | FG | Plastic octopus pots | 0 | 0.0 |
| J70 |  | Plastic shotgun cartridges | 0 | 0.0 |
| J11 |  | Plastic beach use related body care and cosmetic bottles and containers | 0 | 0.0 |
| J12 |  | Plastic non-beach use related body care and cosmetic bottles and containers | 0 | 0.0 |
| J95 | SUP | Plastic cotton bud sticks | 0 | 0.0 |
| J29 |  | Plastic combs/hair brushes/sunglasses | 0 | 0.0 |
| J70 |  | Plastic shotgun cartridges | 0 | 0.0 |
| J98 |  | Plastic diapers/nappies | 0 | 0.0 |
| J236 | SUP | Other plastic personal hygiene and care items | 0 | 0.0 |
| J96 | SUP | Plastic sanitary towels/panty liners/backing strips | 0 | 0.0 |
| J144 |  | Plastic tampons and tampon applicators | 0 | 0.0 |
| J97 | SUP | Plastic toilet fresheners | 0 | 0.0 |
| J237 |  | Plastic wet wipes | 0 | 0.0 |
| J253 |  | Plastic single-use face-mask | 0 | 0.0 |
| J211 |  | Other plastic medical items (swabs, bandaging, adhesive plasters etc.) | 0 | 0.0 |
| J100 * |  | Plastic medical/pharmaceuticals containers/tubes/packaging | 0 | 0.0 |
| J99 |  | Plastic syringes/needles | 0 | 0.0 |
| J9 |  | Plastic bottles and containers of cleaning products | 0 | 0.0 |
| J15 |  | Plastic engine oil bottles & containers >50cm | 0 | 0.0 |
| J14 |  | Plastic engine oil bottles & containers <50 | 0 | 0.0 |
| J17 |  | Plastic injection gun containers/cartridges | 0 | 0.0 |
| J16 |  | Plastic jerry cans | 7 | 6.0 |
| J22 * |  | Plastic caps/lids chemicals, detergents (non-food) | 0 | 0.0 |
| J23 * |  | Plastic caps/lids unidentified | 0 | 0.0 |
| J24 * |  | Plastic rings from bottle caps/lids | 0 | 0.0 |
| J13 |  | Other plastic bottles & containers (drums) | 3 | 4.5 |
| J3 | SUP | Plastic shopping/carrier/grocery bags | 122 | 0.7 |
| J101 |  | Plastic dog/pet faeces bag | 0 | 0.0 |
| J5 | SUP | The part that remains from tear-off plastic bags | 0 | 0.0 |
| J36 |  | Other plastic heavy-duty sacks | 0 | 0.0 |
| J238 |  | Plastic mesh bags for vegetable, fruit and other products | 0 | 0.0 |
| J4 | SUP | Small plastic bags | 0 | 0.0 |
| J91 * |  | Plastic mass holder from sewage treatment plants and aquaculture | 0 | 0.0 |
| J18 |  | Plastic crates, boxes, baskets | 0 | 0.0 |
| J65 |  | Plastic buckets | 1 | 0.2 |
| J93 |  | Plastic cable ties | 0 | 0.0 |
| J84 |  | Plastic cds & dvds | 0 | 0.0 |
| J67 |  | Plastic sheets, industrial packaging, sheeting | 0 | 0.0 |
| J225 |  | Plastic food containers made of hard non-foamed plastic | 3 | 1.7 |
| J64 |  | Plastic fenders | 3 | 3.0 |
| J68 |  | Fibreglass items | 0 | 0.0 |
| J63 |  | Plastic floats/buoys other source than fishing or not known | 1 | 1.2 |
| J239 |  | Other foamed plastic items and fragments not made of foamed polystyrene | 1 | 0.7 |
| J257* |  | Foamed plastic packaging | 0 | 0.0 |
| J83 |  | Fragments of foamed polystyrene >50cm | 0 | 0.0 |
| J82 |  | Fragments of foamed polystyrene <50 | 0 | 0.0 |
| J80 |  | Fragments of non-foamed plastic >50cm | 39 | 87.5 |
| J79 |  | Fragments of non-foamed polystyrene <50 | 0 | 0.0 |
| J240 |  | Other identifiable foamed plastic items | 0 | 0.0 |
| J241 |  | Other identifiable non-foamed plastic items | 3 | 1.4 |
| J166 |  | Plastic paint brushes | 0 | 0.0 |
| J28 |  | Plastic pens and pen lids | 0 | 0.0 |
| J49 |  | Plastic rope (diameter more than 1cm) | 11 | 3.5 |
| J242 |  | Plastic string and cord (diameter less than 1cm) not from dolly ropes or unidentified | 3 | 0.1 |
| J66 |  | Plastic strapping bands | 0 | 0.0 |
| J43 |  | Plastic tags (fishing, shipping, farming and industry) | 0 | 0.0 |
| J87 |  | Plastic masking/duct/packing tape | 1 | 0.0 |
| J88 |  | Telephone | 2 | 0.8 |
| J72 |  | Plastic traffic cones | 0 | 0.0 |
| J86 |  | Plastic fin trees (from fins for scuba diving) | 0 | 0.0 |
| J243 |  | Plastic remains of fireworks | 0 | 0.0 |
| J32 * |  | Plastic toys and party poppers | 0 | 0.0 |
| J27 * | SUP | Tobacco products with filters (cigarette butts with filters) | 0 | 0.0 |
| J26 |  | Plastic cigarette lighters | 0 | 0.0 |
| J25 |  | Plastic tobacco pouches/plastic cigarette packet packaging | 0 | 0.0 |
| J19 |  | Plastic vehicle parts | 3 | 28.5 |
|  |  | **Rubber** |  | 0.0 |
| J127 |  | Rubber boots | 0 | 0.0 |
| J133 |  | Rubber condoms (incl. Packaging) | 0 | 0.0 |
| J131 * |  | Rubber band (small, for kitchen/household/post use) | 0 | 0.3 |
| J248 |  | Rubber sheet | 0 | 0.0 |
| J134 |  | Other rubber pieces | 5 | 3.1 |
| J249 |  | Rubber belts | 0 | 0.0 |
| J125 * | SUP | Rubber balloons | 0 | 0.0 |
| J126 |  | Rubber balls | 0 | 0.0 |
| J250 |  | Rubber inner-tubes | 2 | 1.9 |
| J251 |  | Rubber tyres | 17 | 407.7 |
|  |  | **Cloth/textile** |  | 0.0 |
| J137 |  | Clothing | 24 | 8.2 |
| J138 |  | Shoes & sandals made of leather and/or textile | 0 | 0.0 |
| J141 |  | Cloth textile carpet & furnishing | 6 | 6.6 |
| J140 |  | Hessian sacks/packaging | 0 | 0.0 |
| J143 |  | Sails, canvas | 0 | 0.0 |
| J145 |  | Other textiles | 5 | 11.4 |
| J139 |  | Cloth textile backpacks & textile bags | 0 | 0.0 |
|  |  | **Paper/cardboard** |  | 0.0 |
| J150 |  | Paper cartons/Tetrapak milk | 1 | 0.0 |
| J151 |  | Paper cartons/Tetrapak (non-milk) | 0 | 0.0 |
| J244 |  | Paper cups | 0 | 0.0 |
| J245 |  | Paper food trays, food wrappers, drink containers | 0 | 0.0 |
| J246 |  | Paper cotton bud sticks | 0 | 0.0 |
| J247 |  | Other paper containers | 0 | 0.0 |
| J147 |  | Paper bags | 0 | 0.0 |
| J148 |  | Cardboard boxes | 1 | 0.2 |
| J156 |  | Paper fragments | 2 | 0.1 |
| J154 |  | Paper newspapers & magazines | 0 | 0.0 |
| J158 |  | Other paper items | 0 | 0.0 |
| J155 |  | Paper tubes and other pieces of fireworks | 0 | 0.0 |
| J152 |  | Paper cigarette packets | 1 | 0.0 |
|  |  | **Processed / worked wood** |  | 0.0 |
| J159 |  | Wooden corks | 0 | 0.0 |
| J165 |  | Wooden ice-cream sticks, chip forks, chopsticks, toothpicks | 0 | 0.0 |
| J164 |  | Wooden fish boxes | 0 | 0.0 |
| J163 |  | Wooden crab/lobster pots | 0 | 0.0 |
| J162 |  | Wooden crates, boxes, baskets for packaging | 0 | 0.0 |
| J172 |  | Other processed wooden items >50cm | 6 | 67.6 |
| J171 |  | Other processed wooden items <50 cm | 0 | 0.0 |
| J160 |  | Wooden pallets | 0 | 0.0 |
| J167 |  | Wooden fireworks & matches | 0 | 0.0 |
|  |  | **Metal** |  | 0.0 |
| J194 |  | Metal cables | 0 | 0.0 |
| J175 |  | Metal drinks cans | 209 | 7.4 |
| J176 |  | Metal food cans | 0 | 0.0 |
| J181 |  | Metal tableware (e.g. Plates, cups & cutlery) | 5 | 0.2 |
| J184 |  | Metal lobster/crab pots | 0 | 0.0 |
| J182 * |  | Metal fisheries related weights/sinkers, and lures | 16 | 253.5 |
| J180 |  | Metal appliances (refrigerators, washers, etc.) | 0 | 0.0 |
| J187 |  | Metal drums & barrels | 0 | 0.0 |
| J174 |  | Metal aerosol/spray cans | 0 | 0.0 |
| J188 |  | Other metal cans | 3 | 0.7 |
| J190 |  | Metal paint tins | 4 | 0.3 |
| J178 * |  | Metal bottle caps, lids & pull tabs from cans | 1 | 0.0 |
| J195 * |  | Metal household batteries | 0 | 0.0 |
| J177 |  | Metal foil wrappers, aluminium foil | 1 | 0.0 |
| J199 |  | Other metal pieces >50cm | 15 | 2535.9 |
| J198 |  | Other metal pieces <50 cm | 3 | 0.5 |
| J186 |  | Metal industrial scrap | 2 | 0.1 |
| J191 |  | Wire, wire mesh, barbed wire | 0 | 0.0 |
| J179 |  | Metal disposable bbqs | 0 | 0.0 |
| J193 |  | Metal vehicle parts/batteries | 1 | 3.7 |
| J130 |  | Wheels with metal hub | 0 | 0.0 |
|  |  | **Glass/ceramics** |  | 0.0 |
| J204 |  | Glass ceramic construction materials (bricks, tiles, cement) | 14 | 212.0 |
| J203 |  | Glass and ceramic tableware (plates/cups/glasses) | 4 | 96.5 |
| J207 |  | Ceramic or glass octopus pots | 0 | 0.0 |
| J200 |  | Glass bottles | 105 | 34.3 |
| J201 |  | Glass jars | 1 | 0.4 |
| J208 |  | Pieces of glass/ceramic (glass or ceramic fragments ≥2.5 cm) | 5 | 0.2 |
| J205 |  | Glass fluorescent light tube | 0 | 0.0 |
| J202 |  | Glass light bulbs | 0 | 0.0 |
| J219 |  | Other ceramic items | 0 | 0.0 |
| J210 |  | Other glass items | 3 | 0.1 |
|  |  | **Chemicals** |  | 0.0 |
| J216 |  | Unidentified generally dark-coloured oil-like chemicals | 0 | 0.0 |
| J217 |  | Unidentified generally light-coloured paraffin-like chemicals | 0 | 0.0 |
| J218 |  | Unidentified chemicals | 0 | 0.0 |
|  |  | **Food waste** |  | 0.0 |
| J215 |  | Organic food waste | 0 | 0.0 |
|  |  | **Other** |  |  |
|  |  | Ancient litter (Amphora) | 3 | 2.3 |

**Table A3.** Two-way PerMANOVA (city and bottom depth) test for difference in litter abundance and mass-based on Euclidean distance resemble matrix. p(MC) is p value of Monte Carlo test.

|  |  | Abundance | | | Mass | | |
| --- | --- | --- | --- | --- | --- | --- | --- |
| Source | df | F | p | P(MC) | F | P | P(MC) |
| Province | 5 | 0.61839 | 0.327 | 0.75 | 0.58972 | 0.389 | 0.79 |
| Depth | 10 | 0.29357 | 0.677 | 1 | 0.96022 | 0.229 | 0.505 |
| Province x Depth | 21 | 1.1133 | 0.272 | 0.292 | 1.497 | 0.156 | **0.038** |
| Residuals | 269 |  |  |  |  |  |  |
| Total | 305 |  |  |  |  |  |  |

(b)

(a)

**Fig A1.** Co-linearity of litter item in abundance distribution classified by province (a) and bottom depth (b)

(b)

(a)

**Fig A2.** Co-linearity of litter item in mass distribution classified by province (a) and bottom depth (b)
